# Cognitive and behavioural strategies for weight management in overweight adults: Results from the Oxford Food and Activity Behaviours (OxFAB) cohort study

**DOI:** 10.1371/journal.pone.0202072

**Published:** 2018-08-10

**Authors:** Jamie Hartmann-Boyce, Paul Aveyard, Carmen Piernas, Constantinos Koshiaris, Carmelo Velardo, Dario Salvi, Susan A. Jebb

**Affiliations:** 1 Nuffield Department of Primary Care Health Sciences, University of Oxford, Radcliffe Primary Care Building, Radcliffe Observatory Quarter, Oxford, United Kingdom; 2 Institute of Biomedical Engineering, Department of Engineering Sciences, Old Road Campus Research Building, University of Oxford, Headington, Oxford, United Kingdom; CUNY, UNITED STATES

## Abstract

**Background:**

Though many overweight and obese adults attempt to lose weight without formal support, little is known about the strategies used in self-directed weight loss attempts. We set out to assess cognitive and behavioural strategies for weight loss and their associations with weight change.

**Methods:**

Prospective, web-based cohort study of overweight UK adults (BMI≥25kg/m^2^) trying to lose weight through behaviour change. Strategy use was assessed using the OxFAB questionnaire and evaluated (1) at the domain level, (2) through exploratory factor analysis, and (3) in a model of strategies deemed *a priori* to be “essential” to weight management. Associations with weight change at 3 months were tested using linear regression.

**Results:**

486 participants answered all questions; 194 reported weight at baseline and at 3 months (mean weight change -3.3kg (SD 4.1)). Greater weight loss was significantly associated with the motivational support domain (-2.4kg, 95% CI -4.4 to -0.4), dietary impulse control (from factor analysis) (-0.6kg, 95% CI -1.1 to -0.03), and weight loss planning and monitoring (from factor analysis) (-1.3kg, 95% CI -2.0 to -0.5). Higher scores in the model of essential behavioural strategies were significantly associated with greater weight loss (compared to participants using 6 or fewer of the 9 strategies, using 7 or more of the 9 strategies was associated with a 2.13kg greater weight loss (SE 0.58, p<0.001)).

**Conclusions:**

Despite heterogeneity in the strategies employed for weight loss, coherent patterns of behaviours emerged for individual participants, some of which were associated with greater weight loss, including strategies relating to dietary impulse control, weight loss planning and monitoring, motivational support, information seeking and self-monitoring. Trials could test the effect of promoting use of these patterns on weight loss.

## Background

Individual level efforts to treat obesity are an important part of a wider population strategy to tackle obesity and reduce the burden of obesity-related diseases. Data from the Health Survey for England show that over 53% of overweight and 76% of adults with obesity living in the UK are currently trying to lose weight.[1] This prevalence is consistent with that reported from other Western countries, with rates of approximately 44% reported among the general population of North American adults.[2] Several systematic reviews have observed that behavioural weight management programmes (BWMPs) designed to support people to lose weight through changes to diet and/or physical activity are effective [3–7], but only a small proportion of people who may benefit currently enrol in formal programmes.[8] Instead, most people trying to manage their weight do so using a range of self-management strategies. Our systematic review showed that, within self-help interventions, the most commonly recommended strategies for weight loss were self-monitoring and goal setting.[5] However, there is a paucity of data on the particular strategies people themselves employ in self-directed weight loss attempts and their effectiveness. This is a critical knowledge gap since evidence of the effectiveness of behavioural interventions is almost entirely derived from strategies that have been shown to be effective in the context of intensive interventions, typically involving multiple contacts with trained staff.

The Oxford Food and Activity Behaviours (OxFAB) taxonomy was specifically developed to categorise the strategies employed by people as part of their weight control efforts.[9] Taxonomies of techniques are increasingly used in behaviour change research, following national and international guidelines, and can be instrumental in establishing a common language and identifying active ingredients of behavioural interventions.[10–12] The OxFAB taxonomy focusses on the behavioural strategies enacted by individuals, rather than the components recommended by interventionists. It has been used to develop a reliable and internally valid questionnaire that participants can use to record their weight management strategies.[9] The OxFAB questionnaire differs from existing questionnaires designed to explore weight-related behaviours, as they primarily focus on quantifying and qualifying energy intake and expenditure (how much and what kind), e.g. DINE [13] or IPAQ [14], measuring the end result of a myriad of behaviours as opposed to the cognitive and behavioural strategies individuals adopt, and which lead to the recorded diet and activity levels.

In this cohort study, we used the OxFAB taxonomy and questionnaire to assess the cognitive and behavioural strategies used for weight change in adults with overweight and obesity attempting to achieve weight loss, and examined the associations between strategies used and weight change after three months of follow-up.

## Materials and methods

### Recruitment and inclusion criteria

The study was approved by the University of Oxford Central University Research Ethics Committee. Recruitment started in January 2015 and this paper presents an analysis of data collected from January to November 2015. Participants were recruited from the general public on a rolling basis, through a number of recruitment channels. Study launch was timed to coincide with a television programme on weight loss airing nationally throughout the UK; the associated website for the television programme contained advertisements for the study, which were the initial driver for recruitment. In addition, the study was publicised on social media, to members of existing studies, and via a local health improvement service. Participants were encouraged to share information on the study with family and friends. The system was open to anyone who wished to register and was over the age of 18, but to be included in the present study, participants were required to be overweight adults (BMI greater than or equal to 25 kg/m^2^, aged 18 or older), resident in the UK, and trying to lose weight through changes to their diet, physical activity, or both. There were no recruitment or retention incentives beyond sharing preliminary findings with participants once the analysis was completed.

### Data collection

A bespoke, cross-platform mobile phone application and a study website (www.oxfab.org) were created to collect data. Participant consent was obtained electronically through the same system. After consenting, participant demographics (age, gender, ethnicity, highest level of education obtained) and self-reported weight and height were collected. Participants were then asked to log in episodically to indicate which specific strategies they were using and to report their weight. Participants could register to receive e-mail prompts to log in to the website on a daily, weekly, or fortnightly basis, or choose to opt out of prompts. If, at three months from baseline, participants had not yet reported a post-baseline weight they were prompted to do so (in order to continue to use the system to log other information). Both the website and the mobile phone application allowed participants to visualise their weight progression on a graph and receive simplified statistics about the strategies they reported using. The data set upon which this analysis is based is publicly available via the Oxford University Research Archive.[15]

### Classification of strategy use

The OxFAB questionnaire was used to establish which strategies were being employed by participants to manage their weight. At baseline, participants were asked three screening questions to rule out questions that were irrelevant to them. Accordingly, participants who were not using diet to control weight were not asked questions specific to diet, participants who were not using physical activity to control their weight were not asked questions specific to physical activity, and participants who did not shop for their own food were not asked questions about food shopping. After irrelevant questions had been screened out, participants were asked all remaining questions from the OxFAB questionnaire in a randomly generated order. This questionnaire consists of 117 questions grouped into 21 domains (see Table 1) and is based on the OxFAB taxonomy, which was constructed through a qualitative analysis of existing resources and a review of existing behaviour change taxonomies and theories. The OxFAB questionnaire has been previously assessed for reliability and validity; details on its development and the full list of questions are available elsewhere.[9] For each question, participants were asked to indicate if they used the strategy most of the time, sometimes, or never/hardly ever. ‘Not relevant’ was also provided as an answer option, and participants had the facility to mark individual questions as unclear. Questions were presented in batches of 10 and participants were given the possibility to continue with the next batch or stop.

**Table 1:** OxFAB taxonomy domains and definitions.

Domain	Definition
Energy compensation	Conscious adjustment of behaviours to alter energy intake and/or expenditure to control weight in light of previous energy intake or expenditure
Goal setting	Setting of specific behavioural or outcome target(s)
Imitation (modelling)	Emulating the physical activity or dieting behaviour of someone who you have observed
Impulse management: Acceptance	Respond to unwanted impulses through awareness and acceptance of the feeling that generates the impulse and reacting without distress or over-analysis
Impulse management: Awareness of motives	Respond to unwanted impulses by evaluating personal motives behind that impulse before acting
Impulse management: Distraction	Respond to unwanted impulses through distraction in an attempt not to act on the impulse
Information seeking	Seek specific information to enhance knowledge to help manage weight
Motivation	Strategies to increase the desire to control weight
Planning content	Plan types of food/physical activity in advance of performing behaviour
Scheduling of diet and activity	Plan timing and context/location of food/physical activity in advance of performing behaviour
Regulation: Allowances	Unrestricted consumption of or access to pre-specified foods or behaviours
Regulation: Restrictions	Avoid or restrict pre-specified foods, behaviours, or settings
Regulation: Rule setting	Mandate responses to specific situations
Restraint	Conscious restriction over the amount that is eaten
Reward	Reinforcement of achievement of specific behaviour or outcome through reward contingent on the meeting of that target
Self-monitoring	Record specific behaviours or outcomes on regular basis
Stimulus control	Alter personal environment such that it is more supportive of target behaviours (adapted from CALO-RE)[14]
Support: Buddying	Perform target behaviours with another person
Support: Motivational	Discussing, pledging, or revealing weight loss goals, plans, or achievements or challenges to others to bolster motivation
Support: Professional	Seek help to manage weight from someone with specific expertise
Weight management aids	Use of and/or purchase of aids to achieve weight loss in any other manner (including, but not limited to, reducing energy intake and increasing energy output)

### Data analysis

Analyses were conducted using Stata v11.[16] Participants were excluded if they had missing data for one or more of the strategies relevant to them or if, when calculated using self-reported weight and height data, their BMI at baseline was less than 25. For analyses of associations between strategy use and weight change at three months, participants were excluded if they had not provided a self-reported weight at 75 to 105 days from baseline. Baseline differences between those who completed all questions and those who did not were calculated using unpaired t-tests for continuous variables and using Pearson’s chi square tests for binary and categorical variables.

In the primary analyses, strategy use was defined dichotomously, with ‘most of the time’ and ‘sometimes’ considered as use of that strategy, and ‘never’ and ‘not relevant to me’ considered as not using that strategy. Domain use was evaluated in two predefined ways: as percentage of strategies used within each domain, and as use of at least one strategy within a domain. These two approaches were used as domains contained varying numbers of strategies.

#### Factor analysis

Data-driven patterns of strategy use were derived by exploratory factor analysis for the previously described binary strategy use variables using a *tetrachoric* correlation matrix with orthogonal rotation (*varimax* option) to enhance factor identification and interpretability. Factors were retained based on eigenvalues >1 and the proportion of variance explained. For each factor identified, every strategy has a factor loading which is the correlation coefficient between each strategy and the factor. Only the strategies with factor loadings (*z-score)*>0.2 were retained and extracted in the factors identified.[17] For each factor identified, factor scores were derived for each participant, with higher scores indicating higher adherence to that factor (e.g. higher usage of the strategies contained in each factor). Factors will be referred as patterns of strategies from now on.

#### Model of essential strategies for weight loss

In addition to the factor analysis, we conducted a non-data driven exploratory analysis testing an *a priori* model which included only those strategies that were deemed essential to weight management. This strategy model was created by the authors based on existing knowledge.[18, 19] It contained nine components: food and weight targets; a strategy to enhance motivation to lose weight; advance meal planning; monitoring of food intake; swapping less healthy foods for healthier ones; keeping unhealthy food out of the house; an impulse management strategy to employ when cues to eat are presented; and self-weighing. Each participant was given a score of 0–9 based on whether or not they used a strategy within that component ‘most of the time’; each component of the model was equally weighted (use of that component = 1).

#### Statistical analysis

A linear multivariable regression model was used to test associations between demographic characteristics and each pattern, and between demographic characteristics and domain use. A linear regression model was used to explore associations between strategy, pattern and domain use and weight at three months controlling for baseline weight.[20] We then tested whether participants’ scores for the model of essential strategies for weight loss were associated with weight at three months controlling for baseline weight using a linear regression model with a continuous scale (0 to 9; with one point for each strategy used within the model) and a binary scale (use of seven or more strategies within this model; this threshold was determined as it represented high use of relevant strategies (mean score 5.4)). For each analysis, all model assumptions for using parametric tests were checked and met. All analyses were exploratory and *p values* should be taken as indicative.

## Results

### Participant characteristics

**Fig 1:**
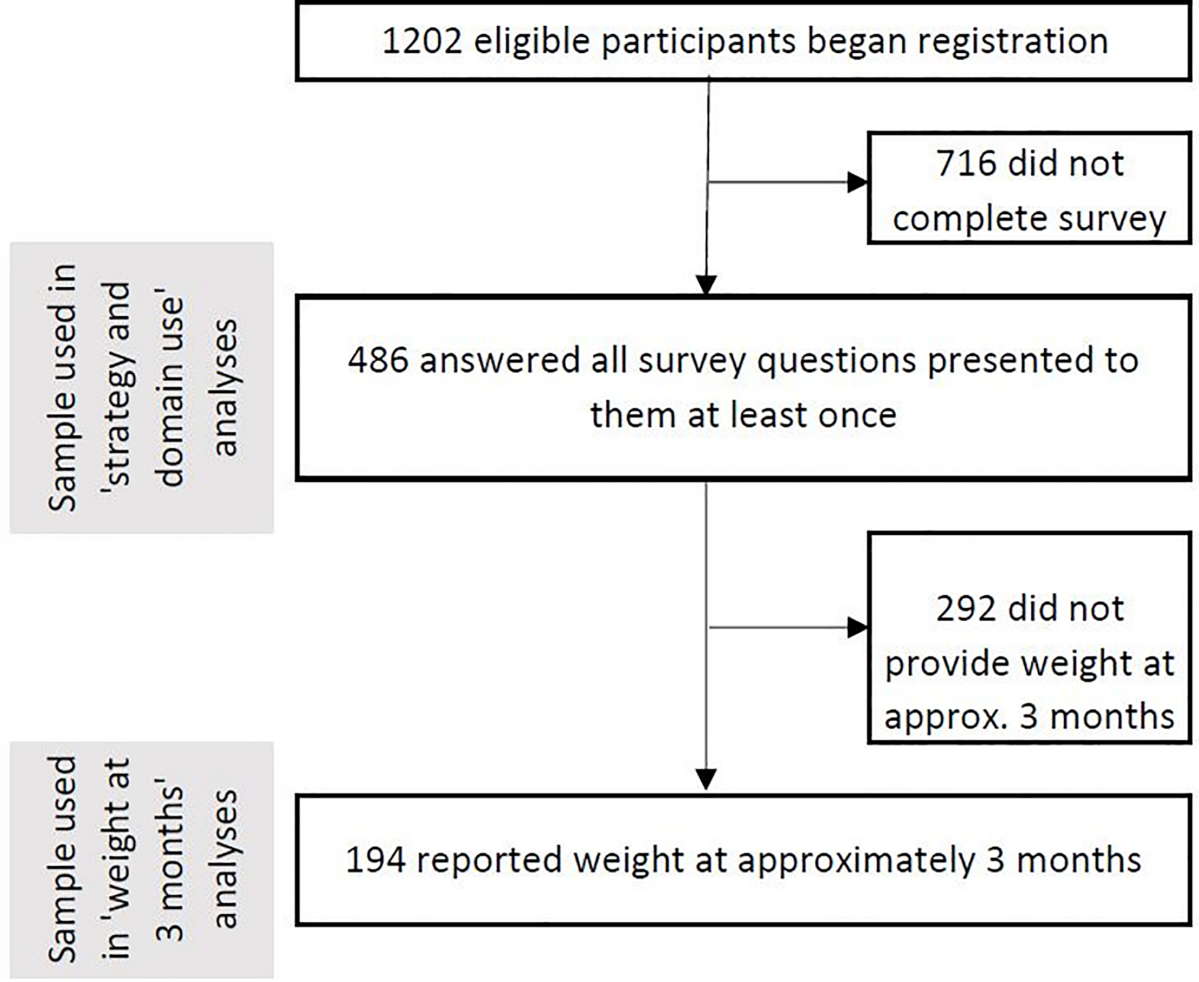
Diagram of study flow.

Four hundred and eighty-six participants answered all survey questions presented to them at least once (survey completers), representing approximately 40% of the eligible participants who began registration (1,202 in total) (Fig 1). Survey completers were significantly more likely to have a university degree than non-completers (*X*^2^ = 21.80, p <0.001), to be of White British/Irish ethnicity (*X*^2^ = 14.41, p = 0.006) and to be older (mean age completers 50.3 (SE 0.69) versus 46.2 (SE 0.65), p<0.001). No other demographic characteristics were associated with survey completion. Baseline demographics for survey completers are reported in Table 2. The majority of participants were White British/Irish with a university degree. The mean BMI at baseline was 33.14 (SD 6.52), and the mean weight was 91.37 kg (SD 18.38). The majority of participants were female (85%).[4] Eighty four percent of participants were attempting to lose weight through changes to both their diet and physical activity, 2% were changing only their physical activity, and 13% were changing only their diet.

**Table 2:** Baseline demographics of survey completers.

Characteristic	Participants (n = 486)	
Gender	414 (85.36%) Female (1 missing)	
Age (mean, SD)	51.81 (11.56)	
Ethnicity	Black British/Caribbean/African	2 (0.41%)
	Mixed and others	33 (6.79%)
	None selected	3 (0.62%)
	South Asian	6 (1.23%)
	White British/Irish	442 (90.95%)
Highest level of education completed	No qualifications	5 (1.03%)
	O/A level or equivalent	129 (26.54%)
	University degree	350 (72.02%)
	None selected	2 (0.41%)
Baseline BMI (mean, SD)	33.14 (6.52)	
Baseline weight (mean, SD)	91.37 kg (18.38)	
Diet and/or exercise to lose weight	Both	407 (84%)
	Exercise only	10 (2%)
	Diet only	65 (13%)
	Missing	4 (1%)
Heard about study	Involved in another weight loss study	21 (4.32%)
	Online advertisement	145 (29.84%)
	Other	299 (61.52%)
	Through a friend/family member	21 (4.32%)

### Strategy and domain use

#### Use of individual strategies

**Fig 2:**
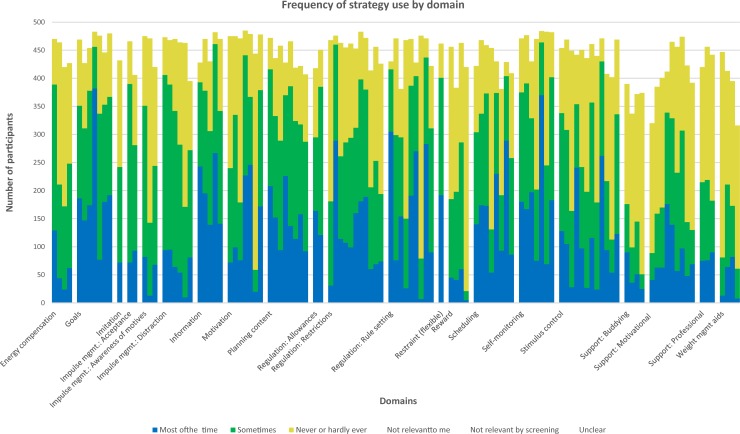
Frequency of strategy use by domain.

On average, participants used 39 (SD 12) strategies some of the time and 29 strategies (SD 14) most of the time (out of 117 strategies in total). The number of participants reporting use of each individual strategy varied considerably (see Fig 2); on average, each strategy was used by 284 participants (58%). Six strategies were used by over 90% of participants at baseline: ‘I keep track of my weight by weighing myself’; ‘I've looked up strategies, tips or plans for how to lose weight’; ‘To help me manage what I eat, I try to avoid eating certain foods’; ‘I have a goal weight in mind that I am working towards’; ‘I keep myself motivated by reminding myself of the reasons I want to lose weight’; and ‘I would swap one type of food or drink for another if I knew one would be better for my diet’.

#### Patterns of strategy use

Factor analysis revealed 5 meaningful patterns of strategy use (Table 3). Patterns were conceptually coherent and we have assigned names for each to aid interpretation.

**Table 3:** Strategy patterns.

Pattern	Name	Groups of strategies
1	Physical activity	I set myself goals for how active I’ll be each day or each week
		I schedule physical activity into my week
		I follow an exercise plan/routine
		I do something to prompt me to exercise (e.g. lay out my exercise clothes the night before)
		If I feel like I want to stop exercising, I focus on something else to distract me so that I keep going
		When I’m being active, I push myself to my limits
		I keep track of the physical activity that I do
		I've chosen a type of physical activity I prefer in order to keep it up
		I give up something else to make time for exercise (e.g. watching TV, socializing, leave work early)
2	Dietary impulse control	I use smaller plates, bowls or glasses when eating to help with my portion control
		When I have a craving for something I shouldn’t eat, I try to think about something else to take my mind off it
		If I feel like eating but am trying not to, I make myself wait a certain amount of time to see if the craving passes
		If I feel like eating but am trying not to, I pause and ask myself if I’m hungry
		Part way through eating a meal, I pause and ask myself if I’m full enough to stop
		I slow down how quickly I eat in an effort to eat less
3	Support	I’ve tried to get the people I live with to encourage my weight loss plans
		I’ve tried to get my friends and family to support me in managing my weight
		I’ve promised other people I'll lose a certain amount of weight
		I belong to a group of people who are trying to lose weight together (for example, an online discussion forum, a group of colleagues all trying to lose weight)
		I am losing weight with a friend/family member/my partner and I'm trying hard to lose more than them
		I feel like I am part of a team with my friend(s)/partner/family member. We are losing weight together.
		I use a weight loss service to help me manage my weight (for example, Weight Watchers, Slimming World, Lighter Life)
4	Advanced dietary planning	When I’m grocery shopping and items of food look similar, I make my choice based on the nutritional information on the food labels
		I plan my food shopping in advance to help me stick to my diet (e.g. use a shopping list)
		To avoid eating or drinking things that don’t fit with my diet, I don’t keep them at home
		When I go food shopping, I try to only buy the types of food that I know I should be eating
		When I am food shopping, there are certain foods I stay away from to help me stick to my diet
		I plan when I am going to do my food shopping to make sure it's not at a time when I am really hungry
5	Weight loss planning and monitoring	I use a chart, graph or diary to track my progress in losing weight
		I use a book, website or app to look up the nutrition information and/or calorie content of the foods I eat
		I have a clear goal for the amount of weight I want to lose each week
		I check the portion sizes of the things I eat
		I have a weight management plan, but I allow myself to be flexible about what I do depending on circumstances

#### Domain use

When defined as use of at least one strategy within that domain, the mean number of domains used per participants was 18.4 (out of 21) (SD 1.84), ranging from 7 to 21 domains. Goals, impulse management: distraction, motivation, restrictions, rule setting, scheduling, self-monitoring and stimulus control were the most commonly used domains (over 95% of participants using at least one strategy within that domain). Table 4 shows the frequency of use of each domain, defined both as average percentage of strategies used within that domain and use of at least one strategy within each domain.

**Table 4:** Frequency of use of each domain.

Domain	Use of at least one strategy within domain		Proportion of strategies used within domain	
(n = 486)	Mean	SD	Mean	SD
Energy compensation	90%	31	52%	32
Goals	100%	6	75%	20
Imitation	50%	50	50%	50
Impulse management: Acceptance	92%	27	69%	31
Impulse management: Awareness of motives	85%	36	51%	31
Impulse management: Distraction	96%	16	64%	25
Information seeking	100%	6	77%	21
Motivation	100%	5	59%	21
Planning content	100%	6	71%	21
Allowances	89%	32	70%	34
Restrictions	99%	9	60%	19
Rule setting	99%	8	64%	18
Restraint	83%	38	83%	38
Reward	81%	39	35%	24
Scheduling	99%	10	58%	22
Self-monitoring	100%	0	71%	20
Stimulus control	99%	10	55%	19
Support: buddying	42%	49	22%	28
Support: motivational	94%	23	41%	23
Support: professional	75%	43	42%	32
Weight management aids	72%	45	27%	22

### Associations between demographics and patterns of strategies and domain use

People with a university degree had on average a 0.23 higher score (95% CI 0.01 to 0.44, p = 0.04) on the physical activity pattern and a 0.31 higher score (95% CI 0.13 to 0.49, p<0.001) on the support pattern than people without a university degree. Men used significantly more strategies than women overall (8.46, 95% CI 4.81 to 12.10, p<0.001) and had significantly higher factor scores for the dietary impulse control pattern (0.43, 95% CI 0.16 to 0.70, p = 0.002), the support pattern (0.49, 95% CI 0.24 to 0.75, p<0.001) and the advanced dietary planning pattern (0.70, 95% CI 0.48 to 0.92, p<0.001). There were no other significant associations between number of strategies used and participant characteristics, or between participant characteristics and factor scores for the weight loss planning and monitoring patterns. At the domain level, men again used significantly more domains than women (0.87, 95% CI 0.42 to 1.32, p<0.001), with no other associations between participant characteristics and number of domains used. Table 5 indicates where there were significant associations between demographic characteristics and levels of domain use.

**Table 5:** Associations between domain use and participant characteristics.

Domain	Significant association when defined as use of at least one strategy within that domain	Significant association when defined as percentage of strategies used within that domain
Energy compensation	University degree (0.07, 95% CI 0.01 to 0.13, p = 0.012) older participants (0.002, 95% CI 0.0001 to 0.004, p = 0.037)	University degree (.08, 95% CI 0.02 to 0.14, p = 0.004)
Goals	None present	None present
Imitation	None present	None present
Impulse management: acceptance	None present	University degree (0.06, 95% CI 0.001 to 0.12, p = 0.024)
Impulse management: awareness	None present	None present
Impulse management: distraction	Younger participants (0.001, 95% CI 0.0002 to 0.002, p = 0.005) men (0.061, 95% CI 0.02 to 0.10, p = 0.002)	Men (0.12, 95% CI 0.06 to 0.18, p<0.001)
Impulse management:	None present	None present
Information	None present	None present
Motivation	Men (0.015, 95% CI 0.001 to 0.034, p = 0.012)	Men (0.06, 95% CI 0.001 to 0.12, p = 0.041)
Planning	White British/Irish (0.009, 95% CI 0.01 to 0.017, p = 0.018)	Men (0.11, 95% CI 0.05 to 0.17, p<0.001)
Allowances	None present	None present
Restrictions	None present	Men (0.08, 95% CI 0.02 to 0.14, p = 0.002)
Rule setting	None present	Men (0.10, 95% CI 0.05 to 0.15, p<0.001)
Restraint	None present	None present
Reward	Men (0.17, 95% CI 0.07 to 0.27, p = 0.001)	Younger age (0.002, 95% CI 0.0001 to 0.003, p = 0.031) men (0.12, 95% CI 0.06 to 0.18, p<0.001)
Scheduling	Men (0.04, 95% CI 0.01 to 0.06, p = 0.005)	Men (0.11, 95% CI 0.05 to 0.16, p<0.001)
Self-monitoring	None present	None present
Stimulus control	Men (0.04, 95% CI 0.01 to 0.06, p = 0.005)	Men (0.10, 95% CI 0.05 to 0.15, p<0.001)
Support: buddying	University education (negative) (-0.15, 95% CI -0.23 to -0.06, p = 0.001)	University education (negative) (-0.07, 95% CI -0.12 to -0.02, p = 0.003)
Support: motivational	None present	Men (0.08, 95% CI 0.02 to 0.14, p = 0.008)
Support: professional	University education (negative) (-0.11, 95% CI -0.18 to -0.03, p = 0.004)	Men (0.10, 95% CI 0.02 to 0.19, p = 0.012) White British/Irish (negative) (-0.04, 95% CI -0.07 to -0.004, p = 0.030) university education (negative) (-0.08, 95% CI -0.14 to -0.03, p = 0.002)
Weight management aids	Younger participants (0.004, 95% CI 0.0009 to 0.006, p = 0.009) men (0.16, 95% CI 0.04 to 0.27, p = 0.007)	Younger people (0.001, 95% CI 0.00005 to 0.003, p = 0.041)

### Weight at three months

One hundred and ninety-four participants reported their weight at approximately three months (75 to 105 days) and are used in subsequent analyses testing associations between strategy or domain use and weight. For this measure, the mean days of follow-up was 89.2 (SD 7.1), and the mean weight change was -3.25 kg (SD 4.07).

### Associations between strategy and domain use and weight

#### Strategies and patterns of strategy use

The number of strategies used at baseline was not significantly associated with weight at three months, when controlling for baseline weight. Two strategy patterns were significantly associated with weight change, with higher factor scores associated with greater weight loss: the dietary impulse control pattern (-0.55 kg, 95% CI -1.08 to -0.03, p = 0.039) and the weight loss planning and monitoring pattern (-1.29 kg, 95% CI -2.04 to -0.54, p = 0.001).

#### Theoretical model of essential strategies

When we tested our model of essential strategies for weight change, the average score was 5.4 out of 9. Using a continuous model, there was a significant association between score and weight change (-0.43 kg, 95% CI -0.69 to -0.18 kg, p = 0.001), as there was using a binary model examining those participants using seven or more strategies most of the time (-2.13 kg, 95% CI -3.27 to -0.99, p<0.001).

#### Domains

The number of domains used at baseline was not significantly associated with weight change. When defined as using at least one strategy within that domain, only one domain, motivational support, was significantly associated with weight change (-2.39 kg, 95% CI -4.39 to -0.39, p = 0.020). In exploratory analyses for strategies within this domain, one was found to be significantly associated with weight change: “I told other people about my weight loss goals to help me stick to them” (-1.16 kg, 95% CI -2.29 to -0.03, p = 0.044). When defining domain use as percentage of strategies used within individual domains, information seeking (-3.93 kg, 95% CI -6.49 to -1.37, p = 0.003) and self-monitoring (-2.89 kg, -5.71 to -0.07, p = 0.045) were both found to be significantly associated with greater weight loss. Within information seeking, exploratory analyses found that using a book, website or app to look up nutritional information was the only strategy significantly associated with weight change (-1.70 kg, 95% CI -3.02 to -0.38, p = 0.012). Within self-monitoring, two strategies were significantly associated with weight change in univariate models: checking portion sizes (-1.50 kg, 95% CI -2.90 to -0.09, p = 0.037) and keeping track of calorie/nutritional content of food consumed (-1.65 kg, 95% CI -2.96 to -0.34, p = 0.014). When combined in a multivariate model, keeping track of calorie and/or nutritional content remained significantly associated with greater weight loss (-1.45 kg, 95% CI -2.78 to -0.12, p = 0.032).

## Discussion

The OxFAB questionnaire was able to identify a large number of frequently used strategies and detect associations between patterns of strategy use and weight change. This analysis reveals large inter-individual differences in the use of specific strategies and groups of strategies (domains). It has identified associations between the use of strategies relating to dietary impulse control, weight loss planning and monitoring, motivational support, information seeking and self-monitoring and greater weight loss at three months. There were also significant associations between weight change at three months and use of strategies in a theory-driven model of those deemed to be essential for weight change. Together this provides useful insights to guide the development of self-management programmes for weight-loss.

This is the first study using the OxFAB taxonomy and cannot be directly compared with other studies, though some related work has been done. The US National Weight Control Registry (NWCR) has evaluated a number of behavioural strategies, but includes only people who have maintained a weight loss of >30 lbs for a year or longer.[21] Though the profile of OxFAB participants is distinctly different from those in the NWCR, and though the NWCR focusses on maintenance as opposed to initial loss, there are important parallels, including findings pointing to the importance of dietary restraint and self-weighing.[22–24] However, strategies that contribute best to weight loss in the shorter term may be different from those that best aid with weight loss maintenance.[18] Outside of cohort studies, results from a large US population based survey in 2006 pointed to the importance of self-monitoring of food intake, but this was a retrospective survey without validated tools for assessing strategy use, and focussed on maintenance rather than initial loss.[25] In terms of those strategies relating to dietary impulse control, a recent systematic review of techniques for modifying impulsive processes associated with unhealthy eating included 92 studies and found promising changes in unhealthy food consumption and food cravings.[26]

This was an exploratory study employing both data driven and *a priori* approaches to examining associations between groups of strategies and weight loss. Both approaches were successful in helping to identify strategies associated with greater weight loss success. In terms of statistical significance of associations detected, p values were smaller for the *a priori* approach (≤0.001], but there was overlap between the strategies identified through both approaches. There were significant associations between greater use of strategies in the *a priori* model and greater weight loss, and factor analysis yielded two patterns associated with greater weight loss: dietary impulse control and weight loss planning and monitoring. Both of these strategy types were also represented in our *a priori* model, and analysis of the association between domains and weight loss also yielded promising results for self-monitoring, as well as for motivational support (also included in our *a priori* model] and information seeking (not included in our *a priori* model]. In summary, some types of strategies were promising across approaches, namely dietary impulse control, self-monitoring, motivational support, and planning. Domain-led analysis also suggested information seeking may contribute to weight loss success.

It is clear that further studies are needed to evaluate links between cognitive and behavioural strategies for weight loss and subsequent weight change, particularly in light of some of the limitations inherent in this study. The sample used in this study is not representative, which limits the applicability of the findings across a range of population groups. In addition, missing data was an issue in the study, which limits the generalisability of our conclusions. However, given the limited number of variables we collected we would be unable to impute the missing data properly, and hence have opted to use a full-case analysis. As an observational study, the OxFAB cohort is unable to show causation, and there may be unmeasured variables which account for associations between strategy use and weight change. However, the types of strategies we found to be associated with weight change have been found to be successful elsewhere, increasing our confidence in the findings. A third limitation of this study is its reliance on self-reported data; the accuracy of self-reported weight is a known issue and is a limitation in all studies of this type.[27] In particular, data suggest that people are likely to underestimate their weight when self-reporting. Reassuringly, self-reported and observed weights have been found to be closely correlated. Two large studies comparing web-based self-reported weight and observed weight recently concluded that self-reported weight loss is comparable with observed.[28, 29]. In this study, both weight at the start and end were self-reported, so there is no reason to assume that weight change is biased. In particular, we judge it unlikely that any bias from self-report weight differs by use or non-use of a particular strategy or group of strategies and therefore data on the association of strategy use and weight change is likely to be valid. A final limitation is that the use of the study website may in itself be considered a strategy to which all participants were exposed.

## Conclusions

Greater weight loss was associated with the use of strategies relating to dietary impulse control, weight loss planning and monitoring, motivational support, information seeking and self-monitoring. There were also significant associations between weight change at three months and use of strategies in a theory-driven model of those deemed to be essential for weight change. These are exploratory findings using a novel methodology and the associations need to be evaluated in future studies. One potential avenue for this would be randomized controlled trials of low-intensity self-help interventions (in order to most closely mimic self-management scenarios) incorporating the most promising strategies, though further observational studies testing associations between cognitive and behavioural strategies and weight change could also prove informative. Further data from such studies could eventually be tested and used to inform the advice and interventions delivered to adults wishing to achieve self-managed weight loss. Adults who are overweight and who are losing weight outside of formal programmes represent a large proportion of the population, and the opportunity to reduce preventable morbidity and mortality amongst this group through evidence based self-help interventions is substantial. Evidence from confirmatory studies using the OxFAB taxonomy would inform the development of such interventions.
